# Cell-type-specific epigenetic effects of early life stress on the brain

**DOI:** 10.1038/s41398-022-02076-9

**Published:** 2022-08-10

**Authors:** Mouly F. Rahman, Patrick O. McGowan

**Affiliations:** 1 Department of Biological Sciences, University of Toronto Scarborough, Toronto, ON Canada; 2 Department of Cell and Systems Biology, University of Toronto, Toronto, ON Canada; 3 Department of Psychology, University of Toronto, Toronto, ON Canada; 4 Department of Physiology, University of Toronto, Toronto, ON Canada

**Keywords:** Epigenetics in the nervous system, Genetics

## Abstract

Early life stress (ELS) induces long-term phenotypic adaptations that contribute to increased vulnerability to a host of neuropsychiatric disorders. Epigenetic mechanisms, including DNA methylation, histone modifications and non-coding RNA, are a proposed link between environmental stressors, alterations in gene expression, and phenotypes. Epigenetic modifications play a primary role in shaping functional differences between cell types and can be modified by environmental perturbations, especially in early development. Together with contributions from genetic variation, epigenetic mechanisms orchestrate patterns of gene expression within specific cell types that contribute to phenotypic variation between individuals. To date, many studies have provided insights into epigenetic changes resulting from ELS. However, most of these studies have examined heterogenous brain tissue, despite evidence of cell-type-specific epigenetic modifications in phenotypes associated with ELS. In this review, we focus on rodent and human studies that have examined epigenetic modifications induced by ELS in select cell types isolated from the brain or associated with genes that have cell-type-restricted expression in neurons, microglia, astrocytes, and oligodendrocytes. Although significant challenges remain, future studies using these approaches can enable important mechanistic insight into the role of epigenetic variation in the effects of ELS on brain function.

## Epigenetics and cellular programming

At the beginning of life, the unicellular fertilized egg, or zygote, gives rise to all the cells of an organism, possessing the property of “totipotency”. As the zygote becomes an embryo, and the embryo a fetus, there is a gradual decline in totipotency of the newly divided cells, narrowing the range of cell types they can become, rendering them “pluripotent”, a function of the plasticity of epigenetic marks. As the embryo develops and cells differentiate, pluripotency declines and epigenetic marks become more stable, determining and maintaining gene expression programs that underlie cell fate. In this way, cellular programming can be defined as the epigenetic process that contributes to stem cell differentiation into mature cell types [1, 2]. As the genetic sequence is virtually identical in all cells within each individual, the epigenome of each cell orchestrates the pattern of gene expression to confer cellular identity through histone modifications, DNA methylation (DNAm), and non-coding RNA (ncRNA).

Octamers of histone proteins coil DNA to form the nucleosomes, which are themselves wound to form chromatin. Modifications of histone N-terminal tails by acetylation, methylation, phosphorylation and ubiquitination play a role in determining DNA accessibility by modifying the positively charged N-terminal tails that tightly interact with the negatively charged DNA. For example, histone acetylation and deacetylation are understood to render chromatin more or less accessible to transcription factors (TFs), leading to enhanced or reduced transcriptional activity, respectively [3]. Histone methylation has also been associated with gene silencing or activation, depending on the amino acid modified [4, 5].

Methylation of DNA is a class of DNA modification that largely occurs at cytosine bases that are followed by guanine bases (CpG sites) in the mammalian genome. Non-CpG methylation, although less frequent, has been found in embryonic stem cells [6], neurons [7] and mature oocytes [8]. At gene promoters, first exons, and first introns, DNA methylation (DNAm) can suppress gene expression by inhibiting TF binding to regulatory elements [9]. DNAm within gene bodies and internal exons involves complex interactions with TF binding sites and conformational chromatin structure [10–12], the regulation of alternative splicing [13, 14], and is positively associated with transcription, especially for ubiquitously expressed genes [12, 15]. DNAm can also suppress gene expression through other mechanisms including histone deacetylase complex recruitment, which introduces histone modifications that result in chromatin silencing [16]. Conversely, TFs themselves can regulate DNAm by binding to specific DNA sequences to protect de novo methylation or recruit DNA methyltransferases to maintain, suppress, or initiate *de novo* DNAm [17].

Several types of ncRNAs, including micro-RNAs (miRs) and long non-coding RNA (lncRNAs) are sometimes considered an epigenetic mechanism due to their prominent roles in epigenetic regulation [18]. miRs are short sequences of nucleotides (~22) that largely repress gene expression post-transcriptionally through complementary binding to their target mRNAs, of which there can be hundreds, prompting the degradation of the corresponding mRNA and ultimately reducing its protein level [19]. The regulation of other genes at the RNA level by miRs is a property shared with other common post-transcriptional regulators of gene expression that are not typically considered part of epigenetics. Other ncRNAs are involved in epigenetic regulation at the nucleus, as is the case for lncRNA [20]. lncRNAs are >100 nucleotides in length, involved in processes including chromatin remodeling, modulation of histone and DNA methylation and acetylation, pre and post-transcription and translation, and have been functionally characterized mainly in the context of cancer [21, 22]. Generally, lncRNAs are processed and operate in the cytoplasm (see review [20]), however, lncRNAs involved in X-chromosome inactivation play a prominent role in the nucleus [22, 23].

The epigenetic program arises in response to fetal environmental signals that include extrinsic and intrinsic signaling molecules and growth factors (see review [24]), genomic imprinting through DNAm and histone modifications, and the DNA sequence itself. Genotypic differences can introduce DNAm sites (e.g. the presence of cytosines) and affect the binding of TFs, which in turn influences epigenetic modifications [17]. Cycles of epigenetic processes involved in genetic imprinting and sex-chromosome dosage compensation also occur in the zygote as discussed [25] and reviewed elsewhere [2, 26]. For example, soon after fertilization, in the pre-implanted embryo, or blastocyst, there is genome-wide demethylation in somatic cells, followed by a global wave of remethylation at implantation with locus-specific changes to methylation that continue into late gestation [27].

**Fig. 1:**
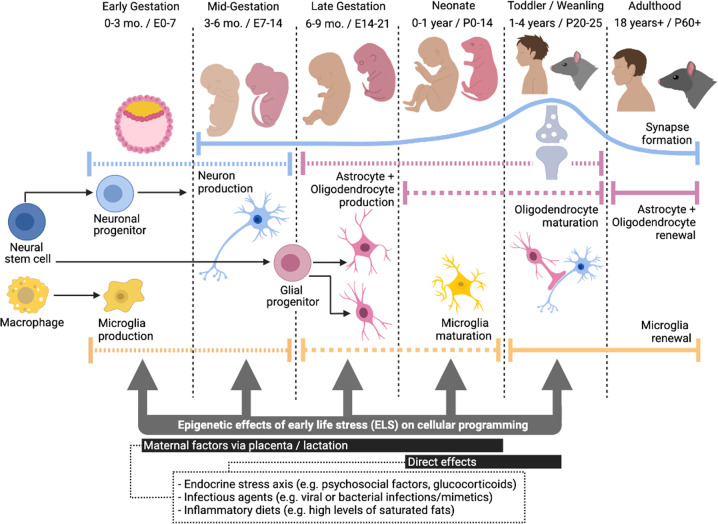
Timeline of cellular programming in the brain and windows of susceptibility for epigenetic effects by early life stress (ELS). Early to mid-gestation, neural stem cells produce neuronal progenitors, precursors for neurons, which migrate and form synapses from mid-gestation to toddler/weanling age. Synaptogenesis starts mid-gestation, peaks in toddler/weanling age, and continues throughout life. In late gestation to toddler/weanling age, neural stem cells give rise to glial progenitors, precursors for astrocytes and oligodendrocytes, which mature from birth to toddler/weanling age. In early and mid-gestation, myeloid-derived macrophages invade the embryonic nervous system to become microglia, which mature into the neonatal period. Microglia and glia continue to have local self-renewal and proliferative capacity into adulthood. Overall, during these periods of cellular production and maturation, ELS factors (e.g. stress hormones, immune stimulants) can alter cell type proportions as well as induce epigenetic reprogramming, leading to long-term changes in the brain. *E* *=* *embryonic day, P* *=* *post-natal day*.

In the embryo, one of the first major phases of cellular programming is the differentiation of the embryonic stem cells into the three germ layers: the endoderm, mesoderm, and ectoderm, the latter of which gives rise to the neuroectoderm, the precursor tissue of the central nervous system (CNS) [28]. The neuroectodermal stem cells, also known as neural stem cells (NSCs), are “multipotent” and produce distinct cell types of the CNS (Fig. 1) [29]. During early gestation, NSCs self-renew and symmetrically divide into two identical daughter cells to increase the pool of multipotent cells. Early to mid-gestation, NSCs divide asymmetrically to produce a NSC and a neuronal progenitor, precursors for neurons. In early and mid-gestation in humans [30–32] and rats [33], myeloid-derived macrophages in the yolk sac, formed from the endoderm [34] invade the embryonic nervous system to become the resident CNS macrophages, or microglia [35, 36], which mature into the neonatal period [37]. In late gestation to toddler age in humans [38, 39] and weanling age in mice [40], NSCs give rise to glial progenitors, precursors for astrocytes and oligodendrocytes [1], with myelination occurring from birth to toddler age in humans [39] and weanling age in rodents [41].

Neurons are largely post-mitotic cells; most production and migration takes place prenatally and continues to a limited degree in the postnatal period [42], with a few neurogenic zones remaining active in adulthood [43]. The formation of synapses starts mid-gestation, peaking at toddler age in humans [44], and weanling age in rodents [42], and continues throughout life. Microglia continue to have local self-renewal and proliferative capacity [35, 36], and glial progenitors continue to proliferate, migrate and mature into adulthood [45]. Neurodevelopment also involves substantial apoptosis and synaptic pruning. Most neuronal cell death occurs early to late gestation, while glial cell death, synaptic pruning and experience-dependent modification occurs mostly at the post-natal stage [29].

### Epigenetic reprogramming by early life stress

Developmental programming is the process by which cellular programming is fine-tuned by environmental factors in fetal and early postnatal stages, referred to as “early life”. This fine-tuning of cellular programs involves epigenetic reprogramming, whereby the epigenetic plan is altered in a persistent manner within a given cell type, can occur in multiple cell types, does not change cell type, and is typically stable across mitotic cell divisions [46, 47]. Epigenetic reprogramming can alter transcript abundance through long-term modifications that persist even in the absence of the initial environmental trigger. Although epigenetic reprogramming can occur later in life, the early life period is more sensitive to stress, or disruptions to homeostasis, since cell fates are established during this time.

Early life stress (ELS) is an acute or chronic factor that takes place at the prenatal, perinatal and/or pre-pubertal postnatal stages and elicits or affects stress responses. The most studied forms of ELS are inflammation-based (e.g. infection, high-fat diets, xenobiotics) and psychosocial (e.g. emotional and physical abuse or neglect, and sexual abuse) [48]. ELS causes elevations in inflammatory cytokines and/or stress hormones that either impact offspring directly or indirectly via the placenta or breastmilk as maternal factors, inducing epigenetic modifications that impact neurodevelopmental trajectories [49]. In humans, inflammation-based and psychosocial ELS factors are most closely associated with neurodevelopmental conditions including autism spectrum disorder and psychiatric disorders related to anxiety, mood and psychosis (schizophrenia) [50–58]. In parallel, evidence from animal studies show that exposure to maternal high-fat diets, immunostimulants such as the bacterial mimic lipopolysaccharide (LPS) and psychosocial stress in early life lead to long-term changes in corresponding behavioral features of these disorders [59–65]. These findings are accompanied by evidence of long-lasting changes in microglial densities, cytokine expression and phagocytic actions involved in synaptic pruning [59, 66, 67], neurotransmission, neural connectivity, and neuron-glia interactions [68–70]. In this context, accumulating evidence indicates that lasting phenotypic changes induced by ELS involve modifications to cellular programs in the brain [57, 71].

Animal models of ELS permit investigation of *in* and ex vivo changes taking place in the brain that are not easily feasible in humans. However, the correspondence to human conditions of ELS may only be approximate. For example, laboratory rodents are immunologically naïve, unlike humans. Additional complications concern the assessment of different forms of psychosocial ELS (e.g. verbal versus physical abuse) and factors known to influence resiliency in humans (e.g. family income or educational attainment) [72, 73] that are difficult to model in animals. Nevertheless, given the complexities of studying the human brain in the context of ELS, rodent models appear necessary to discover causal pathways and enable the development of molecular targets for therapeutics.

### Epigenetic reprogramming by ELS: the current landscape

**Fig. 2:**
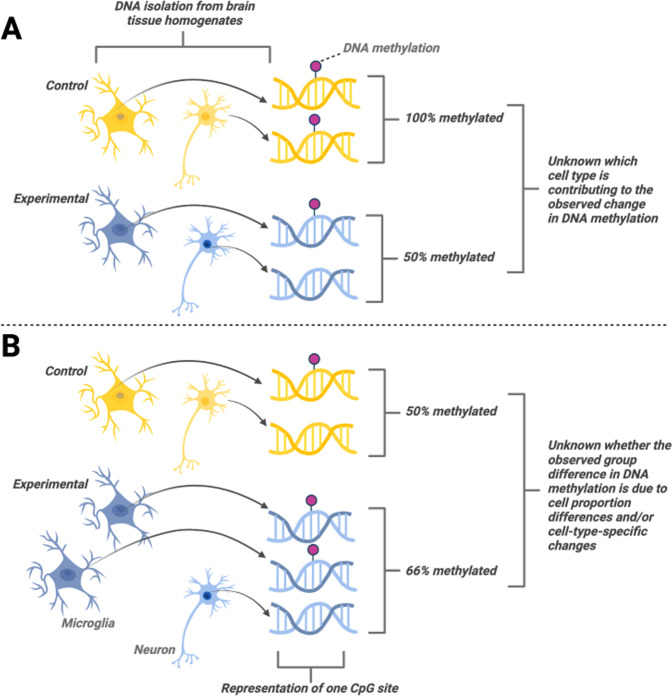
Limitations of examining heterogenous brain tissue for measuring epigenetic modifications. **A** Group differences in the epigenetic mark of a particular gene are not indicative of which cell type(s) are responsible for driving these changes. **B** Group differences in the epigenetic mark of a particular gene may be masked by differences in cell type proportions between groups.

The conventional approach to studying epigenetic reprogramming by ELS involves quantifying epigenetic modifications to select genes, or whole genomes, in heterogenous brain tissue as reviewed elsewhere [48, 49, 74–77]. One classic finding is changes of levels of hippocampal DNAm in the promoter region of the glucocorticoid receptor (*Nr3c1;* GR), a gene linked to impaired negative feedback inhibition of the hypothalamic-pituitary-adrenal (HPA) axis [78], in response to ELS (e.g. childhood abuse, neglect). However, studies of heterogenous brain tissue pose limitations on defining the mechanistic roles of such epigenetic modifications (Fig. 2). First, differences in the epigenetic mark (and expression levels) of a particular gene that is expressed in multiple cell types may be difficult to interpret if the function of a given gene varies between cell types. Second, differences in the epigenetic modifications of a gene expressed in multiple cell types can be masked by ‘background noise’ resulting from constitutive epigenetic modifications related to cell type-specific functions. Returning to the previous example, GR is expressed in all cell types in the brain [79], thus levels of DNAm of the *Nr3c1* promoter in heterogenous brain tissue are not indicative of which cell types are driving such changes. GR can exert distinct genomic and rapid non-genomic functions in different cell types, regulating inflammation in glia [80, 81] and excitability in neurons [82], for example. Furthermore, perturbations in early development can influence cell fate determination, leading to differences in cell type proportions within tissues between experimental groups [83]. For example, neonatal maternal separation has been found to deplete oligodendrocyte progenitors in adult male mice [84]. Epigenetic modifications (e.g., DNAm) measured in particular genes in heterogenous tissue could then be reflective of cell type proportion differences rather than epigenetic reprogramming of a particular cell type(s). To mitigate these potential confounds, some recent studies have examined cell-type-specific epigenetic modifications.

Here we review studies that have examined the effects of ELS on epigenetic modifications in cell-type-enriched isolates, or in genes from heterogenous brain tissue where expression is known to be restricted to a single cell type in the brain (Table 1). We considered ELS studies that are inflammation-based or psychosocial that took place at the prenatal (maternal gestational), perinatal, or neonatal stages of development in female and male offspring aged post-natal day (PND)1 to adulthood. We considered both human and rodent studies, although only one human study was found within our scope. As our focus is on ELS, we did not review effects on offspring due to pre-conceptional parental or transgenerational stress as reviewed elsewhere in heterogeneous brain tissue samples [85–88]. In the brain, the primary categorization of cell types includes neurons, glia (microglia, astrocytes, oligodendrocytes), pericytes and brain epithelial cells [89, 90], however cells in these broad categories can be subdivided based on cellular functions. For example, neurons can be classified as being either glutamatergic or GABA-ergic, excitatory or inhibitory, as well as by cortical layer of origin [91–93]. For the purposes of this review, and consistent with available data in the literature, the primary categorization for cell type was used.

**Table 1:** Summary of reviewed articles whereby epigenetic modifications of genes from cell-type-enriched isolates, or genes with cell-type-enriched expression from heterogenous brain tissue, were measured in offspring exposed to early life stress.

Cell type	Analysis	Stress timing	Animal	Early life stress type	Age point(s)	Sex	Brain region	Findings	Reference
Neuron	Isolates	Postnatal	Rat	Maternal separation	PND56	Male	DG	↑ Retinoic acid receptor promoter DNAm of CpG island	[102]
				Neonatal stress	PND90	Both	mPFC	↑ *Bdnf* IV DNAm across 12 CpG sites (females only)	[100]
	Enriched expression	Prenatal	Rat	Maternal stress	PND0	Male	Whole brain	↑ miR-323	[108]
				Maternal stress	PND60	Male	HPC & PFC	↑ miR-133b in HPC ↓ miR-133b in PFC	[103]
		Postnatal	Mouse	Maternal separation	PND42–56	Both	AMY	↑ *Htr1a* promoter DNAm (females)	[113]
				Maternal separation	PND16	Male	HPC	↑ *Arc* promoter histone H4ac	[109]
			Rat	Maternal separation	PND60	Both	PVN & CeA	↓ *Crh* promoter CpG1 and CpG2 methylation in PVN	[119]
				Maternal separation and/or CUS	Adult	Male	NAc & striatum	↑ miR-326 in NAc ↓ miR-326 in striatum (for CUS & MS+CUS only)	[117]
				Maternal separation	Adult	Undeclared	Ca1	*↑ Crh* promoter histone H3ac *↓ Crh* promoter CpG methylation	[64]
Microglia	Isolates	Prenatal	Mouse	Maternal allergic asthma	PND35	Female	Whole brain	WGBS, DMR, functional annotation, pathway analysis	[129]
		Postnatal	Rat	Maternal separation+ handling	~PND60	Male	NAc	↓ *Il10* gene CpG island methylation	[131]
				Neonatal alcohol	PND6 & 90	Combined	Mediobasal hypoT	↑ *Tnfa* promoter histone H3K9ac ↑ *Il6* promoter histone H3K9ac	[130]
	Enriched expression	Prenatal	Rat	Maternal LPS	PND1	Undeclared	Whole brain	↓ miR-146 & miR-126	[132]
Oligoden-drocyte	Isolates	Postnatal	Human	Childhood abuse	Childhood abuse: 19–85 years Controls: 15–81 years	Combined	ACC	↓ *LINGO3* gene CpG methylation ↓ *POU3F1* gene CpG methylation	[139]
	Enriched expression	Prenatal	Rat	Maternal stress	PND0	Male	Whole brain	↑ miR-219	[108]
Astrocyte	Enriched expression	Postnatal	Rat	Maternal separation	PND35	Male	Frontal cortex	↓ *Gfap* promoter CpG methylation	[138]

*ac* acetylation, *ACC* anterior cingulate cortex, *AMY* amygdala, *Arc* activity-regulated cytoskeleton-associated protein, *Bdnf* brain derived neurotrophic factor, *CeA* central amygdala, *Crh* corticotropin releasing hormone, *CUS* chronic unpredictable stress, *DG* dentate gyrus, *DNAm* DNA methylation, *DMR* differentially methylated regions, *Gfap* glial fibrillary acidic protein, *HPC* hippocampus, *Htr1a* serotonin receptor 1A, *HypoT* hypothalamus, *Il10* interleukin 10, *LINGO3* leucine-rich repeat and immunoglobulin-like domain-containing nogo receptor-interacting protein 3*, LPS* lipopolysaccharide, *miR* micro-RNA, *mPFC* medial prefrontal cortex, *MS* maternal separation, *NAc* nucleus accumbens, *PND* postnatal day, *POU3F1 POU class 3 homeobox 1*, *PVN* paraventricular nucleus of the hypothalamus, *WGBS* whole genome bisulfite sequencing.

### Epigenetic modifications induced by ELS in neurons

In the adult human brain, there is approximately a 1:1 ratio of neurons to glia, although this varies across brain regions [94, 95], similar to rodents [96]. Epigenetic alterations to particular neurons as a consequence of ELS can alter phenotypes associated with neural circuits involved in the response to stress, fear memory, and cognition [97–99]. Dysregulation or impairments of related behaviors are associated with epigenomic alterations in the brain in response to ELS. For example, there are differences in DNAm modifications of genes regulating the HPA axis, monoamines, and neuropeptides in humans with childhood trauma and in animals exposed to ELS [77, 97, 99]. However, it remains unclear whether neurons are ultimately the cell types driving the observed epigenetic changes.

To date, only a few studies have isolated neurons from rodent models of ELS to assess epigenetic modifications. One study found that an adverse caregiving environment from PND1–7 led to increased brain-derived neurotrophic factor (*Bdnf*) exon IV DNAm across 12 CpG sites in neurons isolated from the medial prefrontal-cortex (PFC) of adult female but not in male rats [100], mirroring findings found in heterogenous PFC tissue [101]. BDNF is involved in learning and memory and is a vital factor in neurodevelopment. Retinoic acid receptor, also critical to neurodevelopment, specifically neural differentiation, was found in another study to have increased promoter CpG island methylation in adult neural precursor cells isolated from the dentate gyrus of adult male rats (females unexamined) that underwent neonatal maternal separation (MS) [102]. While additional findings are needed to characterize the impacts of ELS on neurons, these studies indicate that neonatal psychosocial stressors may lead to aberrant gene expression programs essential for neural development.

Some studies that have used whole brain tissue to measure ELS-induced epigenetic changes have measured levels of miRs or DNAm of genes that are only expressed in a single cell type. In adult male rats born to dams with gestational psychosocial stress, miR-133b was found to have increased levels in the hippocampus (HPC) and decreased levels in the PFC [103]. miR-133b shows neuron-enriched expression in the brain [104, 105] and is involved in promoting neurite outgrowth [106]. The same ELS in another study in adult male rats was found to increase whole brain levels of the neuron-specific miR-323 [107], which is involved in host-pathogen interactions with viruses [108]. Together, these findings provide evidence that gestational ELS induces neuron-specific epigenetic reprogramming associated with neuronal growth and immune regulation.

Effects of postnatal stress on the neuronal epigenome have mostly been examined through MS paradigms, whereby neonatal rodents are typically separated from their dam for 3–4 hours/day from PND1–10 or 16. In one study, at PND16 immediately after MS, male mice were found to have increased hippocampal histone H4 acetylation at the promoter of activity-regulated cytoskeleton-associated protein (*Arc*) [109], which is only expressed in neurons and plays a critical role in learning and experience-induced synaptic plasticity [110, 111]. In adulthood, in the amygdala of female but not male mice, MS led to increased DNAm at the promoter of the neuron-specific serotonin-1A receptor (*Htr1a*) [112], which modulates emotional behavior [113]. MiR-326, which has neuron-enriched expression in the neocortex [105, 114, 115] and targets the dopamine D2 receptor [116] was found to be increased in the nucleus accumbens (NAc) and reduced in the striatum of adult male rats exposed to MS alone, and MS combined with maternal chronic unpredictable stress [117]. Also in adult rats (sex undeclared) with neonatal MS, there was increased hippocampal CA1 histone H3 acetylation and decreased methylation of CpGs in the promoter of the neuron-specific [118] corticotropin releasing hormone (*Crh*) [64]. Similar to the latter study, which also found evidence indicative of increased CRH expression, another study in adult rats of both sexes exposed to MS showed reduced *Crh* promoter CpG1 and CpG2 methylation, but in the paraventricular nucleus (PVN) of the hypothalamus [119]. PVN secretion of CRH kickstarts the HPA axis stress response, while CRH binding in the HPC is primarily responsible for regulating glutamatergic transmission and memory function [120]. Altogether, these findings suggest that MS leads to long-term alterations to emotion and learning processes via epigenetic reprogramming of neurotransmitter receptors and neurohormones.

### Epigenetic modifications induced by ELS in microglia

Microglia comprise about 5–12% of the total glia population in the CNS in adult humans [121] and rodents [122] depending on the brain region. In resting states, microglia are involved in synaptic remodeling, maintenance and monitoring of the CNS environment with cell surface receptors that bind to antigens, antibodies, cytokines, and hormones. When potential insults are recognized, such as infection, inflammation, and neurodegeneration, microglia become more ‘activated’, altering their morphology to become phagocytic and releasing inflammatory cytokines that alert neighboring cells and influence their functioning [123]. Towards the end of the insult, microglia release anti-inflammatory cytokines and phagocytose cellular debris. When the insult diminishes, microglia return to their ‘resting’ state. Potentiated and dysregulated states of microglial inflammation are harmful to the tissue environment and can kill healthy neurons. Aberrant microglial activation has been associated with epigenetic dysregulation in anxiety, mood and autism spectrum disorders [124, 125] as well as neurodegenerative diseases [126, 127]. Notably, a similar phenotype indicative of exaggerated inflammation has been found in studies examining epigenetic effects of ELS in animal models [49, 108]. However, these studies utilized heterogenous brain tissue to form their epigenetic links. In addition to microglia, inflammation-related genes including cytokines are expressed by neurons, astrocytes and pericytes [128], making it difficult to determine the extent to which microglia drive neuroinflammatory changes in response to ELS.

Few studies have documented microglia-specific epigenetic changes in response to ELS. In microglia isolated from the whole brain of adolescent female mice subject to maternal allergic asthma exposure, whole-genome-bisulfite sequencing of differentially-methylated regions showed enrichment of gene sets associated with cytokine signaling pathways [129]. In microglia isolated from the medial hypothalamus of PND6 and adult rats (sexes combined), neonatal alcohol exposure was found to increase acetylation of histone H3K9 at the promoter regions of the pro-inflammatory cytokines tumor necrosis factor alpha (*Tnfa*) and interleukin (*Il*)6 in baseline conditions and 2 h post-LPS challenge [130]. Similar to these findings that suggest there is an increased pro-inflammatory phenotype in offspring exposed to ELS, the anti-inflammatory cytokine *Il10* showed reduced levels of CpG island methylation in microglia isolated from the NAc of adult male rats that underwent MS as well as handling, a change that was absent in whole tissue from the NAc [131]. In the whole brain of PND1 rats (sex undeclared) subject to maternal LPS exposure 48 h earlier, there was reduced miR-126 and miR-146 [132], which regulate microglial inflammatory processes [107]. Since changes to both pro- and anti-inflammatory cytokines have been observed in these cell-type-specific studies, future studies can delineate how inflammatory pathways in microglia are epigenetically reprogrammed in response to ELS.

### Epigenetic modifications induced by ELS in astrocytes and oligodendrocytes

In the human cerebral cortex, the glial population includes approximately 20% astrocytes and 75% oligodendrocytes [133], densities of which have also been examined in various brain regions in mice [134]. Astrocytes play critical roles in maintenance of homeostasis through ion buffering, immune signaling, blood-brain-barrier maintenance, regulation of neuronal synaptogenesis and removal [135]. Epigenetic dysregulation of astrocytes and reduced astrocyte cell proportions have been linked to psychiatric disorders associated with ELS, including major depressive disorder [136]. One study found that maternal separation led to reduced promoter CpG methylation of glial fibrillary acidic protein *(Gfap)*, an intermediate filament protein expressed only in astrocytes [137], in the frontal cortex of adolescent male rats [138]. There is also some evidence of ELS-induced epigenetic reprogramming of oligodendrocytes, the myelinating cells of the CNS that enable saltatory nerve conduction and axon integrity. Isolated oligodendrocytes from the anterior cingulate cortex of human suicide completers with a history of childhood abuse exhibited decreased CpG methylation of myelination-regulating genes, specifically, leucine-rich repeat and immunoglobulin-like domain-containing nogo receptor- interacting protein 3 (*LINGO3*) and POU class 3 homeobox 1 (*POU3F1*), compared to controls who died of non-suicide causes [139]. An opposite effect was observed in male rats exposed to maternal gestational psychosocial stress, where there were increased levels of whole-brain miR-219 [108], which shows oligodendrocyte-enriched expression [107] and is necessary for enabling and promoting the maturation of oligodendrocyte precursor cells into myelinating oligodendrocytes [140, 141].

### Challenges and outlook

Converging evidence supports the role of epigenetic modifications as mediators of the impact of ELS on long-term neurobiological alterations. Unlike genetic factors, the epigenome is potentially dynamic throughout life. This malleable quality of the epigenome may enable the identification of cell-specific epigenetic biomarkers of disease and aid in the development of targeted therapeutic approaches for neuropsychiatric or neurodevelopmental disorders, as is the case for certain cancers [142]. Examples of these developments include epigenetic reprogramming of rodent neuronal stem cells to ameliorate neurodegeneration [143] and de-methylation of the fragile X mental retardation (*FMR*) gene in human neurons derived from Fragile X syndrome patients to normalize neuronal activity [144]. Possible epigenetic treatments for neuropsychiatric conditions have also been reviewed elsewhere [145–147].

The study of cell-type-specific epigenetic modifications requires sophisticated technical approaches. Isolating a purified cell population with intact DNA/RNA/protein from the brain can necessitate large sample sizes and volumes of fresh tissue, requiring tools such as primary cell culture, density-based centrifugation, magnetic bead separation, flow cytometry, and single-cell sequencing. These methods require advanced expertise and may be difficult to access. While computational deconvolution tools exists for obtaining cell-type-specific information from bulk sequence reads from heterogenous tissue, they nonetheless provide a less accurate estimation [148].

Of course, epigenetic changes found in a certain cell type do not necessarily mean that similar changes are absent in other cell types. While measuring epigenetic changes in one cell type may provide information regarding mechanisms associated with its biological function, measuring them in multiple cell types would be needed to assess the specificity of such modifications. Absent the heroic effort of quantifying all major cell types within a brain region of interest, comparing epigenetic changes in a single cell type to those of heterogenous tissue is another potential strategy. In such a context, two types of information would be needed: 1) information pertaining to the epigenetic modification of the particular cell type (e.g., microglia) compared to whole tissue from the brain region of interest, and 2) quantification of the proportion of the cell type of interest relative to other cell types in the region of interest. This information would indicate both relative levels of the epigenetic modification in the cell type in the brain region of interest and cell proportion differences that may also contribute to the overall levels of the epigenetic modification (see Fig. 2). This could be particularly important in interpreting the observed epigenetic profiles, which may result from changes in the relative levels of the epigenetic modification in the cell type of interest (potentially indicating cellular reprogramming) and/or in the proportion of the cell type of interest within the region of interest. Notably, potential cell-proportion differences *alone* may constitute an epigenetically-determined phenotype [83, 84].

Based on the available literature, it is evident that cell-specific epigenetic changes in glia are presently very limited in the context of ELS studies, making this fertile ground for future discovery. As well, measurement of ncRNA has been limited to miRNA in the context of the analysis of cell-specific epigenetic changes due to ELS. Given the prominent role of lncRNA in cell type differentiation in the brain [149], and its association with ELS [150] and stress-related disease [22], cell-type-specific changes in lncRNA levels in the context of ELS is another important area for future inquiry.

Studying epigenetic modifications at a primary cell-specific resolution, while important, does not evade interpretational issues. As noted above, beyond the broad categorization of cell types in the brain, definitions of specific cell types are still contested [151]. Neurons can be further subdivided into categories that consider molecular, morphological, connectional, and functional properties, leading to conceptual difficulty in defining a cell type and a lack of a consensus on their taxonomy [152, 153]. Microglia are known to exist in a resting or an activation state, which can be divided into M1 and M2 states of activation that define the bounds of a spectrum of intermediate phenotypes that can vary according to where in the brain the microglia reside [123]. Thus, it is not guaranteed that similar epigenetic modifications will exist within defined cell types, even within a brain region. While single-cell sequencing can alleviate this problem by examining cells at an individual level, this does not seem to solve the problem entirely. To draw an example from cancer biology, histone methylation differences can exist among the same cell types at the center and periphery of the tumor [154]. As such, isolating particular cells involved in a ‘neural circuit’ or biological pathway may be more informative in delineating epigenetic changes contributing to particular phenotypes.

Analysis of the temporal dynamics of epigenetic modifications may also provide important information. Epigenetic modifications detected in temporal proximity to environmental factors are not always the same as the ones detected later. For example, one study found that in the PFC of female rats exposed to neonatal caregiver maltreatment, there was reduced *Bdnf* exon IV methylation at adolescence compared to normal care controls, but an increase at adulthood, and no differences at infancy [101]. Therefore, the temporal dynamics of epigenetic responses to environmental factors that lead to persistent effects on phenotypes is another important avenue for future research.

Another challenge concerns the interpretation of observed epigenetic modifications in relation to their role in gene expression and behavioral phenotypes. For example, such a relationship may not manifest as steady-state increases/reductions in transcript and may only be detected in specific conditions, such as after stress exposure. A study described earlier demonstrates this possibility; neonatal alcohol exposure was found to increase histone acetylation at *Tnfa* and *Il6* promoters in hypothalamic microglia in adult rats at baseline and post-LPS challenge, however increased transcript abundance of microglial mRNA of these genes only occurred in the post-LPS condition and not at baseline [130]. Interpretational challenges also extend to determining whether epigenetic modifications at select loci are ‘causal’ mechanisms of behavior, which may only be possible by altering target epigenetic modifications in vivo [155–157]. Tools used for cell-type-specific epigenetic editing include zinc-finger proteins, which can be fused with histone modifiers and target specific DNA sequences, and transcriptional activator-like effectors (TALEs), DNA binding proteins from bacteria that can be targeted to regulate gene transcription (see review [156]). More recent tools to alter epigenetic modifications include the CRISPR (clustered regularly interspaced short palindromic repeat)/Cas9 system, whereby the prokaryotic RNA-guided endonuclease can be targeted to a specific genomic locus using designed single guide RNA, and a catalytically dead (dCas9) is used to avoid genetic double-strand breaks [155]. The cell-type-specific expression of epigenetic modifiers (e.g., histone acetylation or methylation proteins) fused to dCas9 are then inducible by Cre recombinase, which can be transgenically or virally co-expressed [155, 156]. A recent study in mice found that dopamine D2 receptor neuron-specific targeting of histone acetylation/methylation at the *Fosb* gene within the NAc led to a phenotype of stress susceptibility or resilience, respectively [158]. Epigenetic editing with CRISPR/dCas9 is not without its limitations, as it can involve off-target effects [155, 159]. However, the use of CRISPR, fusion and DNA binding proteins in combination with cell-specific analysis may help delineate the epigenetic mechanisms through which ELS leads to long-term perturbations on behavior, and improve therapeutic approaches for a variety of neuropsychiatric and neurodevelopmental disorders.
